# Accelerated iron valence cycling in Fenton system using activated hydrogen enhanced by MIL-100(Fe) modified by nano-Pd0 Particle

**DOI:** 10.55730/1300-0527.3615

**Published:** 2023-06-23

**Authors:** Yun-Dong WANG, Yi-Jun CHEN, Mei-Na CHENG, Xin LIU

**Affiliations:** 1 Institute of Environmental Protection Application Technology, School of Environmental Science and Engineering, Jiangsu Collaborative Innovation Center of Technology and Material of Water Treatment, Suzhou University of Science and Technology, Suzhou, Jiangsu Province, China; 2 Suzhou Kete Environmental Protection Co., Ltd., Suzhou, Jiangsu Province, China

**Keywords:** Fenton reaction, active hydrogen, iron valence cycling, MIL-100(Fe), nano-Pd^0^ particle

## Abstract

In this paper, a novel Fenton reaction system which was called MHACF-MIL-100(Fe) was constructed. In this system, based on active hydrogen-accelerated Fe^III^ reduction, the hydroxyl radical was continuously produced with a trace amount of total iron. The MIL-100(Fe) modified with the nano-Pd^0^ particle could be used to activate the H_2_. Under normal temperature and pressure, the target organic pollutants, such as sulfamethazine and 4-chloro phenol, could be degraded fast. In the condition of initial aqueous solution pH 3, 2 g L^−1^ dosage of MIL-100(Fe) catalyst loaded with nano-Pd^0^, Pd/MIL-100(Fe), 20 mM 30 wt% hydrogen peroxide, 25 μM ferrous chloride and 60 mL H_2_ min^−1^, 97.8% of sulfamethazine and 100% 4-chloro phenol could be degraded within only 5 min, respectively. Although the surface of the catalyst exhibited more obvious defects and roughness after 5 consecutive destructive experiment cycles, its basic structure could be maintained. The removal efficiency could be maintained at least more than 79% (sulfamethazine) and 94% (4-chloro phenol). That may be mainly attributed to the degradation of hydroxyl radical.

## 1. Introduction

Due to its easy availability of raw materials, easily controlled operation conditions and high mineralization efficiency, the classical Fenton reaction is widely used for the degradation of organics which are difficult to biodegrade. The hydroxyl radical (•OH), which has superb oxidative properties, is the core of this reaction [1].

When the Fenton reaction is finished, the pH of the aqueous solution needs to be regulated in time to meet the discharge requirements. However, the iron sludge will appear in the aqueous solution at once. That may be attributed that the ferrous salt is incessantly added into the system to maintain the concentration of •OH because of the fast consumption rate of Fe^II^[2]. The solid waste could not be properly disposed of. Therefore, the further application of the Fenton reaction is limited [2].

Reducing the usage of ferrous can radically reduce the iron sludge. Accelerating the reduction of Fe^III^ is a feasible strategy. In previous studies, the reduction of Fe^III^ could be promoted by energy input such as light [3], electricity [4], and sound [5], though the maximum energy utilization of less than 40% was not practicable. In addition, the reduction of Fe^III^ can also be significantly promoted by the reducing organics, such as N, N’-ethylenediaminodisuccinic acid (EDDS) [6], 2-methoxyhydroquinone [7], 3,4,5-trihydroxy benzoic acid [8], though this is not easy to control precisely and there are problems with secondary contamination.

Hydrogen is a clean and efficient inorganic reducing agent. In previous studies [2], it was used to accelerate the reduction of ferric. H_2_ could be adsorbed and activated by Pd^0^ nanoparticle [9]. Only less than 2 mg L^−1^ of total iron was required to continuously mineralize methyl tert-butyl ether (MTBE). Since H_2_ is insoluble in water at room temperature, the whole system needs to be confined and pressurized to improve the utilization of H_2_. If the loading of Pd^0^ could be increased, it is obvious that the requirement for hydrogen partial pressure can be reduced, though significantly it was not economical. Therefore, how to increase the utilization of hydrogen in the condition of low usage of Pd^0^ is central to this research direction. The metal-organic frameworks (MOFs) materials may be one of a good substitute for the nano-Pd^0^.

Metal organic backbone (MOFs) materials are three-dimensional crystalline materials generated by the coordination of metal centers and organic ligands. In recent years, MOFs materials have received wide attention in the fields of chemical catalysis, gas storage, gas adsorption and conversion, drug delivery, environmental monitoring, and environmental remediation. Metal-centered Fe is nontoxic. And the material with large specific surface area is hydrothermally stable. It could be used in adsorption, photocatalysis, and Fenton-like applications. This materials, with high porosity and surface area, could be used for the hydrogen storage [10]. And it is also a natural platform for loading Pd^0^ particles [11]. After it was used in the hydrogen-accelerated Fenton system mentioned above, a novel system called MOFs-H_2_-accelerated-catalytic-Fenton (MHACF) was constructed. The typical refractory organics such as sulfamethazine (SMT) and 4-chloro phenol (4-CP) could be eliminated rapidly [11]. Although a series of MOFs materials modified by nano-Pd^0^ particle were used in this MHACF system such as MIL-101(Cr)[11], NH_2_-MIL-101(Cr) [12], MOF-808(Zr) [13] and UiO-66(Zr) [14], continuing to try to find stable MOFs material is still a worthwhile exploration. This is of great significance for expanding the application fields of MOFs materials and developing new advanced oxidation processes.

In previous study, it was reported that the MIL-100(Fe) exhibited stability when it was used as a solid catalyst in Fenton-like reaction [15]. Therefore, it was used to construct the MHACF system in this work. Both refractory SMT and 4-CP were used as target pollutants. The main research objectives of this paper were (1) to achieve the recycling of trace iron; (2) to verify the efficient oxidation capacity of the novel MHACF system; (3) to test the durability of MIL-100(Fe).

## 2. Materials and methods

### 2.1. Chemicals and materials

1,3,5-benzene tricarboxylic acid and sodium borohydride were purchased from Aladdin Chemical (Shanghai, China). Palladium dichloride, ferrous chloride, polyvinyl alcohol (PVA), hydrogen peroxide (H_2_O_2_, 30 wt%), ethanol, HNO_3_, HCl, hydrofluoric acid, and sodium hydroxide were purchased from China Pharmaceutical Corporation (Shanghai, China). All solutions used in the experiments were prepared with Sartorius (Arium comfort I (Germany). All the chemicals were of analytical purity and were not further purified.

### 2.2. Analysis methods

The concentration of target organic pollutants and 4-hydroxybenzoic acid (p-HBA) was analyzed using an LC-20AT high-performance liquid chromatograph (Shimadzu, Japan). A 150-mm column named ZORBAX Eclipse XDB-C_18_ was selected. The specific analysis conditions were listed in Table. The detection methods of Fe^II^, H_2_O_2_ and NH_4_^+^ were from previous works. The concentration of inorganic anions such as inorganic NO_2_^−^, NO_3_^−^, and SO_4_^2−^ was determined by ICS-900 ion chromatography (Thermo, USA). The Multi NC 3100 TOC meter (Jena, Germany) was used to analyze the total organic carbon (TOC). The organic intermediates were analyzed by HPLC-MS equipped with the same column mentioned above. There were two kinds of solutions that were used as mobile phase. One was 2.0% glacial acetic acid solution, the other was acetonitrile. The analysis conditions were as follows: In the condition of 0.4 mL min^−1^, 10% acetonitrile was changed to 60% in 10 min, then was changed to 90% in 8 min, finally was changed to 10% in 10 min. The MS section uses an ESI with a positive ion detection mode.

### 2.3. Synthesis of MIL-100(Fe)

First, 1.03 g of 1,3,5-benzenetricarboxylic acid and 0.41 g of 200-mesh reduced iron powder were thoroughly mixed in 40 mL of pure water at room temperature. Then 0.57 mL of HNO_3_ and 0.3 mL of hydrofluoric acid were added. After it was stirred for 1 h, the mixture was transferred into a 100-mL reaction kettle which was lined with PTFE. The kettle was placed in a blast drying oven for 1 day in the condition of 150 °C. The crystalline orange-red solid was washed with pure water at 70 °C for 5.5 h, followed by C_2_H_5_OH at 60 °C for 3 h, and finally with pure water at 70 °C for 3 h. After the material was filtered and vacuum-dried at 70 °C for 0.5 day, the original MIL-100(Fe) material was obtained. It was activated at 200 °C for 0.5 day before used in reaction [17].

### 2.4. Synthesis of Pd/MIL-100(Fe)

First, 0.0094 g polyvinyl alcohol was dissolved in an appropriate amount of water. The mixture was added into a two-necked flask. Then 21.9 mL of 100 mg L^−1^ PdCl_2_ was added to solution. The mixture was stirred in an ice-water bath for 1 h. Then 1.2 mL NaBH_4_ solution which concentration was 0.5 M was added into the two-necked flask. The pH was adjusted to 7–8 by NaOH solution and HCl solution. Then 0.5 g of the activated MIL-100(Fe) was added and stirred for 4.5 h. The solid obtained after filtering was reactivated in a vacuum-drying oven at 200 °C [18]. That was the preparation of Pd/UiO-66(Zr) (Pd 0.507 wt% based on ICP-OES).

### 2.5. Operation of the MHACF-MIL-100(Fe) system

A 150-mL double-necked flat-bottomed flask was placed in a fume hood at atmospheric pressure and temperature (20 ± 2 °C). The H_2_, ferrous and solid catalyst was successively supplied into the aqueous solution. After adding hydrogen peroxide into the aqueous solution, the reaction started timing. The reaction was sampled using a disposable syringe. The mixture was filtered by 0.45 μm polyethersulfone filter. The filtrate was placed in a liquid sample bottle prefilled with 1 drop of methanol. This methanol was used to burst any reactive oxygen particles that may remain in the filtrate.

### 2.6. Durability test of MHACF-MIL-100(Fe) system

One hundred and eighty minutes was set as a complete reaction cycle. At the end of each cycle, the material was recovered by centrifugation for 5 min at 6000 r min^−1^ using a high-speed centrifuge (TGL-16G Jiangsu Xinkang Medical Devices Co., Ltd.). The supernatant was then decanted. And the collected material was dried in a vacuum-drying oven at 105 °C for 3 h.

### 2.7. Characterization of the MOFs material modified by nano-Pd^0^ particle

The Brunauer-Emmett-Teller (BET) surface area of the solid powder was analyzed placed 77 K by N_2_ adsorption and desorption experiments using a BET tester after the material had been degassed under vacuum at 378 K for 1 day. The functional groups of the MOFs materials were determined in this experiment using Fourier transform infrared (FT-IR) spectroscopy infrared spectrograms using the KBr press method with the FT-IR test range set at 400–4000 cm^−1^. A D8 Advance X-ray diffractometer (Bruker, Germany) was selected to analyze the crystalline phase of the powder at CuKa radiation emission and 40 kV/40 mA current. The crystal morphologies and particle size of the samples were observed by a FEI Quanta FEG 250 scanning electron microscope (SEM, Thermo Fisher, USA). The surface morphologies of the samples were observed by a FEI Tecnai G2 F30 S-TWIN high-resolution transmission electron microscope (HRTEM, Thermo Fisher, USA) equipped with energy-dispersive X-ray analysis (EDXA, USA). The surface morphologies of the samples were observed by a FEI Tecnai G2 F30 S-TWIN high-resolution transmission electron microscope (HRTEM, Thermo Fisher, USA) equipped with energy-dispersive X-ray analysis (EDXA, USA). The determination of TCr and TPd content in the material was carried out by ICP-OES (Agilent 720ES, Santa Clara, California, USA). The content of Pd in the fresh prepared Pd/MIL-100(Fe) was 0.507 wt%.

## 3. Results and discussion

### 3.1. Characterization of materials

**Figure 1:**
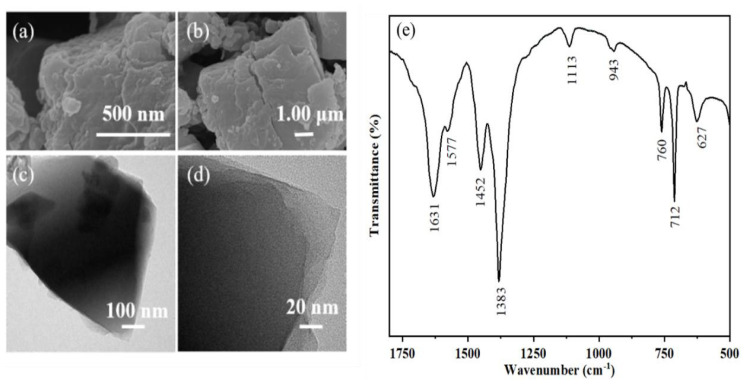
Characterization of MIL-100(Fe): (a) the SEM photograph (500 nm scale); (b) the SEM photograph (1 μm scale); (c) the TEM photograph (100 nm scale); (d) the TEM photograph (20 nm scale) ; (e) FT-IR spectrum of MIL-100(Fe).

The SEM images shown in Figures 1a and 1b revealed that the surface structure of the synthesized MIL-100(Fe) was rough. As shown in the TEM images in Figures 1c and 1d, the MIL-100(Fe) nanoparticles exhibited a polyhedral shape [19]. As seen in Figure 1e, due to the substitution of the terminal ligand -X by methyl-containing groups upon vacuum activation at 200 °C, the peaks of 1452 cm^−1^ and 1383 cm^−1^ were derived from the deformation vibrations of methyl groups on the organic backbone [20]. Those peaks of 760 cm^−1^ and 712 cm^−1^ may be attributed to Fe-OH vibrations [21]. Those peaks of 1113 cm^−1^ and 943 cm^−1^ may be related to the stretching vibrations of C-O. Those peaks of 1577 cm^−1^ and 1631 cm^−1^ may be attributed to the vibrations of double bonds on aromatic rings [22].

**Figure 2:**
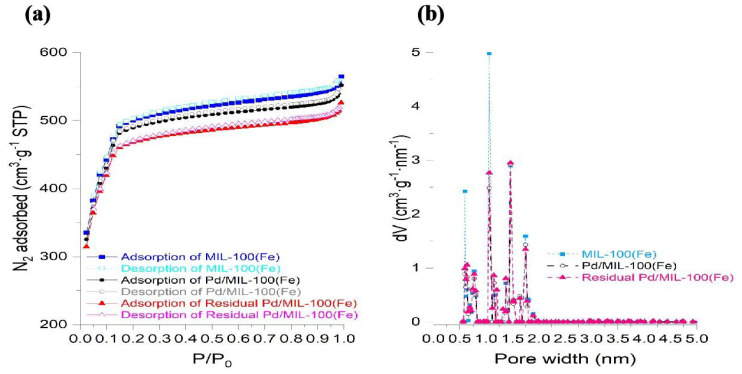
(a) N_2_ adsorption-desorption curve, (b) BET pore size distribution.

**Figure 3:**
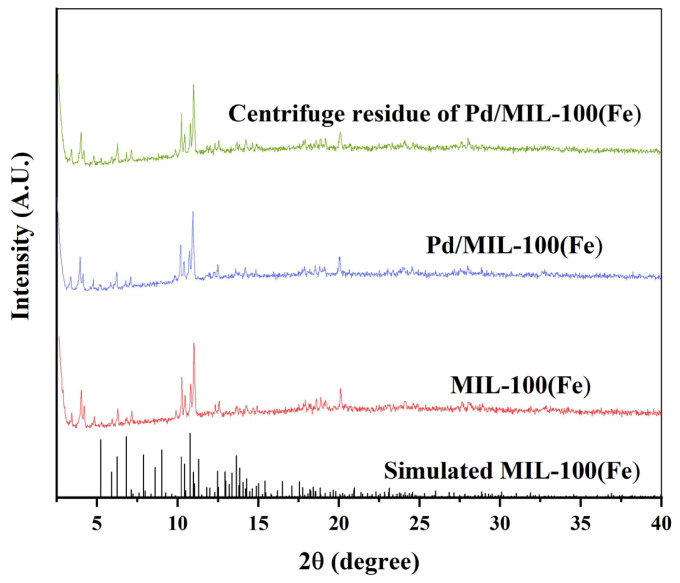
XRD spectra of MIL-100(Fe), Pd/MIL-100(Fe) and Pd/MIL-100(Fe) after five consecutive reaction cycles.

The pore size distribution and surface area of MIL-100(Fe), determined using the N_2_ adsorption-desorption method, were shown in Figure 2. Its specific surface area was 1982.60 m^2^ g^−1^. And the pore size of the material was approximately 0.5–1.8 nm, demonstrating that this material is microporous. It was similar to the previously reported result [23]. As seen the XRD patterns of MIL-100(Fe) shown in Figure 3, the characteristic 2θ values of 6.29°, 10.25°, 11.01°, 12.58°, 18.61°, and 20.10° were corresponded to the (333), (660), (428), (1022), (7911), and (4814) crystal planes, respectively [24]. In combination with the above characterization results, the MIL-100(Fe) material was successfully synthesized.

**Figure 4:**
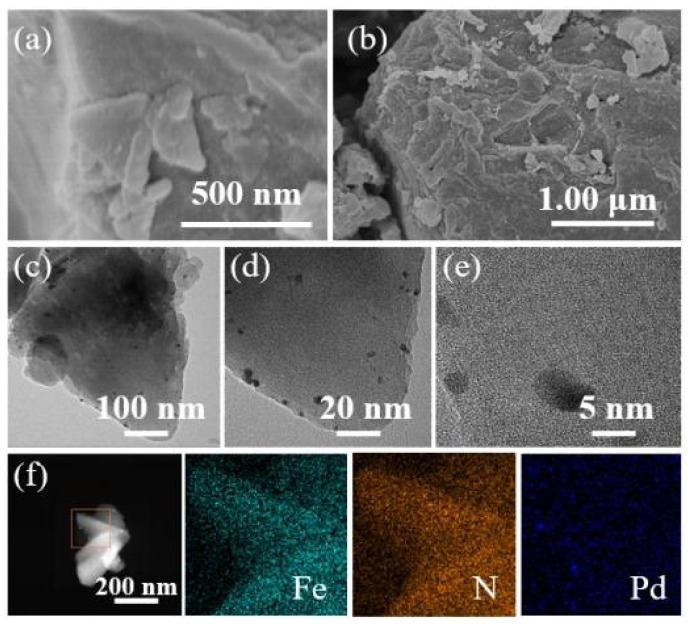
(a) SEM photograph (500 nm scale), (b) SEM photograph (1 μm scale), (c) TEM photograph (100 nm scale), (d) the TEM photograph (20 nm scale), (e) the TEM photograph (5 nm scale) and (f) element mappings images of Pd/MIL-100(Fe).

Also as seen in Figure 2, the specific surface area of as-prepared Pd/MIL-100(Fe) was slightly reduced to about 1180 m^2^ g^−1^. It is obvious from the TEM displayed in Figure 4 that the Pd^0^ nanoparticles with a particle size of about 7 nm are uniformly attached to the surface of the MIL-100(Fe) material. And there were no significant changes in the pore size distribution. Evidently, the nano-Pd^0^ particle with a particle size of about 7 nm could not be loaded into the channels of MIL-100(Fe) with a maximum pore size of 1.8 nm. Although the Pd^0^ occupied part of the surface area of MIL-100(Fe), the specific surface area and pore size distribution were not significantly changed due to its extremely low loading. As seen in the XRD patterns exhibited in Figure 3, the crystal structure of Pd/MIL-100(Fe) was similar to that of MIL-100(Fe). The nanoparticles of Pd^0^ are loaded on the surface of the material with a particle size up to about 15 nm, and their encapsulation tends to have irregular morphology, which reduces the crystallinity of the material [25]. As shown in Figures 4a–4e, the basic structure of MIL-100(Fe) could be saved after the nano-Pd^0^ particle was loaded. As shown in Figure 4f, both Fe and Pd could be detected [26]. Combining the results of the above characterization, it can be seen that the nano-Pd^0^ particles were successfully loaded on the surface of MIL-100(Fe).

### 3.2. Conversion of iron valence during the reaction

**Figure 5:**
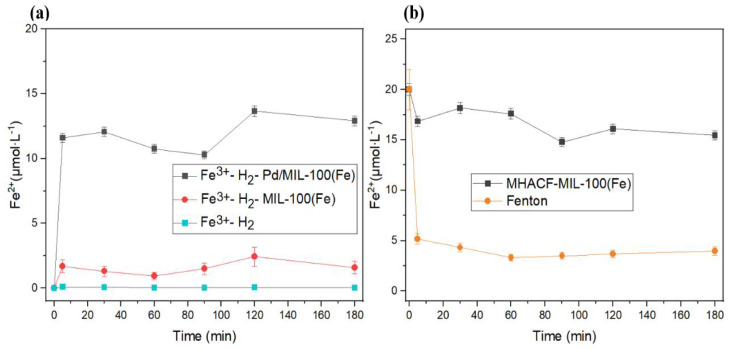
Variation of ferrous in (a) Fe^3+^-H_2_-Pd/MIL-100(Fe), Fe^3+^-H_2_-MIL-100(Fe) and Fe^3+^-H_2_ system with an initial aqueous pH of about 2.5; (b) MHACF-MIL-100(Fe) and Fenton system. Experimental conditions Fe^3+^ 20 μmol L^−1^, initial pH 3, Pd/MIL-100(Fe) 2 g L^−1^, H_2_O_2_ 20 mmol L^−1^ and H_2_ 70 mL min^−1^.

The reduction of ferric was conducted in Figure 5. As shown in Figure 5a, without using solid catalyst, the ferrous was not detected. In systems without the addition of solid catalyst, Fe^2+^ was not detected. The reason for this phenomenon may be that the solubility of H_2_ in water at room temperature and pressure is only 1.83% [27]. Most of the H_2_ returned into the atmosphere at once. Even if some hydrogen could be dissolved in water, it cannot be reduced because it cannot be activated. Conversely, no matter if MIL-100(Fe) or Pd/MIL-100(Fe) was used, H_2_ could be activated. The ferric could be reduced by the generated active hydrogen. In the H_2_-MIL-100(Fe) system, the concentration of ferrous could be 2.4 μM. In the H_2_-Pd/MIL-100(Fe) system, the concentration of ferrous could be 13.6 μM. The H_2_ could be activated better after the introduction of nano-Pd^0^ particle (Equation (1)). As shown in Figure 5b, the concentration of ferrous decreased from 20 μM to 5.17 μM after 5 min of reaction in the classic Fenton reaction. Only 25.9% of TFe was accounted for after 3 h of reaction. In contrast, the ferrous could be maintained at least 72% of TFe in the novel system. That may be attributed that the introduction of hydrogen significantly delayed the accumulation of ferric in the Fenton reaction.

### 3.3. The reactive oxygen species in the novel system

**Figure 6:**
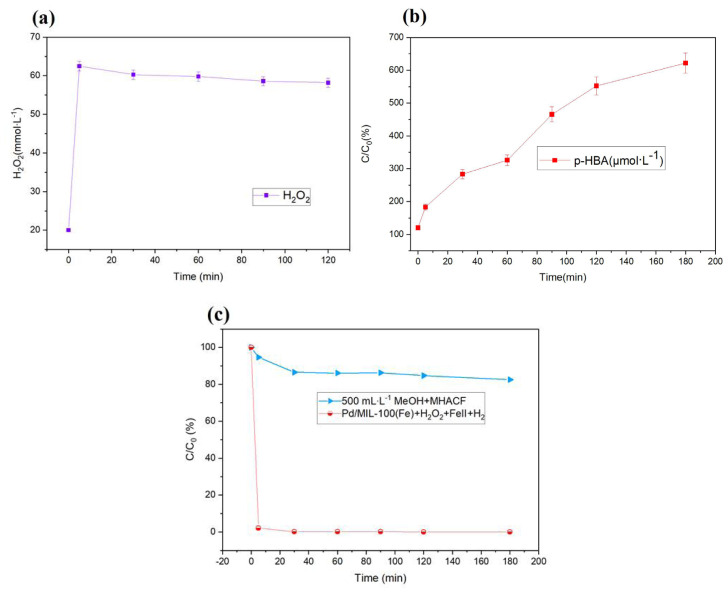
(a) Variation of H_2_O_2_ in the MHACF- MIL-100(Fe) system; (b) concentration of p-HBA generated in the MHACF-MIL-100(Fe) system; (c) inhibitory effect of methanol on the degradation of SMT. Experimental conditions: Fe^2+^ 20 μmol L^−1^, H_2_O_2_ 20 mmol L^−1^, pH 3.0, Pd/MIL-100(Fe) 2 g L^−1^, H_2_ 60 mL min^−1^, SMT 20 mg L^−1^, BA 1.81 g L^−1^ and MeOH 500 mL L^−1^.

As the reaction proceeded, the concentration of H_2_O_2_ increased quickly from 20 μM to 62.5 μM in the MHACF system, as shown in Figure 6a. It could be attributed to the combination of hydrogen and O_2_ catalyzed by zero-valent palladium (Equation (3)) [28]. However, the H_2_O_2_ decreased slightly to 59.8 mmol L^−1^ in the next time. The H_2_O_2_ could be consumed by Fe^2+^, Pd^0^, and MIL-100(Fe), respectively [29].

As shown in Figure 6b, the BA was used as a trapping agent for •OH[16]. The p-HBA concentration increased from 0 μM to 552.5 μM. This indicated that •OH existed in the MHACF system.

The SMT was used as target pollutant. It is a common refractory antibiotic. According to previous works [30], the coagulation of conventional ferrous salts, the direct oxidation of H_2_O_2_ and the simple blowing and reduction of H_2_ have an almost negligible effect on the removal of SMT. It could be degraded by •OH [31]. After the addition of CH_3_OH used as a quencher of hydroxyl radical [31], as shown in Figure 6c, the removal efficiency of SMT was just about 18%. Compared with that of MHACF system, the removal of SMT was significantly inhibited. It was the •OH that dominated the degradation of pollutants in this system. This was consistent with the results reported in previous works [11].


(1) $$\text{Pd}-\text{H}+{\text{Fe}}^{\text{III}}\to {\text{H}}^{+}+{\text{Fe}}^{\text{II}}+{\text{Pd}}^{0}$$

(2) $$\text{FeII}+{\text{H}}_{2}{\text{O}}_{2}\to \text{FeIII}+\text{OH}-+•\text{OH }(\text{the classic Fenton reaction})$$

(3) $${\text{O}}_{2}+{\text{H}}_{2}\overset{\text{Pd}}{\to }{\text{H}}_{2}{\text{O}}_{2}$$

(4) $${\text{H}}_{2}{\text{O}}_{2}\overset{\text{Pd}}{\to }2•\text{OH}$$

### 3.4. Degradation of refractory sulfamethazine in the System

**Figure 7:**
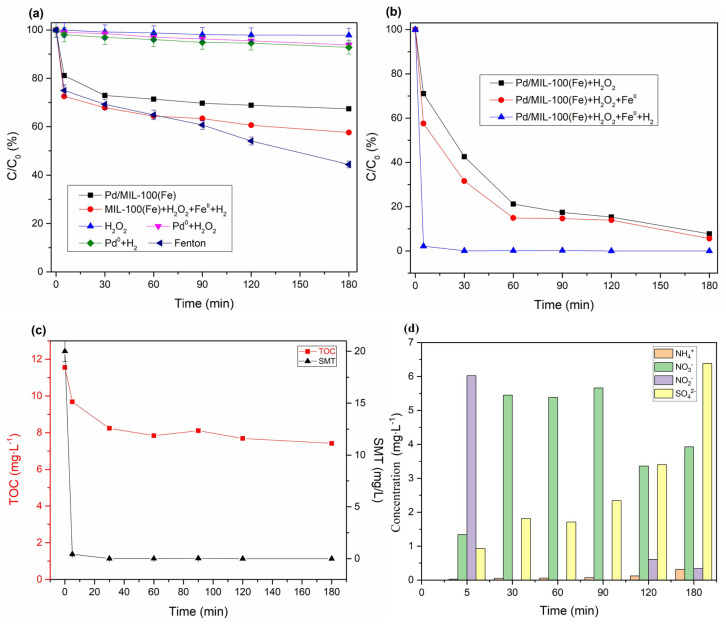
(a–b) The control tests; (c) variation of TOC and SMT, and (d) inorganic ions in the MHACF-MIL-100(Fe) system. Experimental conditions: Fe^2+^ 24.6 μmol L^−1^, H_2_O_2_ 20 mmol L^−1^, pH 3.0, Pd/MIL-100(Fe) 2 g L^−1^, Pd^0^ 0.0098 g L^−1^, H_2_ 70 mL min^−1^ and SMT 20 mg L^−1^.

As shown in Figure 7a, when replacing Pd/MIL-100 (Fe) with MIL-100 (Fe), the removal efficiency within 180 min was only 42.4%, most of which came from the adsorption of the material. Only putting Pd^0^ into SMT solution did not degrade the pollutants. The Pd/MIL-100(Fe) powder could adsorb 32.6% of SMT. The oxidizing property of H_2_O_2_ contributed only 2.2% removal efficiency of SMT. After the introduction of a trace amount of nano-Pd^0^ particle into the mixture of H_2_O_2_ and SMT, the removal efficiency of SMT increased to 6.2% based on the generation of •OH derived from the decomposition of H_2_O_2_ catalyzed by Pd^0^ (Equation (4)) [32]. Seven and two-tenths percent of SMT could be removed in the Pd^0^+ H_2_+O_2_ system by Fenton-like reaction because of the combination of hydrogen and oxygen catalyzed by Pd^0^ (Equation (3)) [33]. In the MIL-100(Fe)+H_2_+Fe^II^+ H_2_O_2_ system, the degradation efficiency of SMT could reach 43.4%. This could be attributed that the Fenton reaction existed in this system. In contrast, a previous study showed that the removal rate of SMT in the UiO-66(Zr)+ H_2_+Fe+ H_2_O_2_ system was only 24.1%, while the removal rate of SMT in the MOF-808(Zr)+ H_2_+Fe+ H_2_O_2_ system was only 29.6%, which was much lower than that of the MHACF-MIL-100(Fe) system [13,14]. This was because it decomposes under the catalysis of Pd^0^ to produce • OH, which oxidizes and degrades SMT. The [H] generated by the activation of H_2_ by Pd^0^ plays an important role in the Fe^III^/Fe^II^ cycle.

Fifty-five and six-tenths percent of SMT was removed by the classical Fenton reaction. As seen in Figure 7b, after the ferrous was substituted by Pd/MIL-100(Fe), 92.3% of SMT could be removed based on the catalytic performance of abundant active site of Fe containing in this material. Furthermore, with the introduction of ferrous ions into the H_2_O_2_+Pd/MIL-100(Fe) system, more •OH could be generated. Therefore, the SMT removal efficiency could be 94.4%. In the Pd/MIL-100(Fe)+H_2_+Fe^II^+H_2_O_2_ system, the degradation efficiency of SMT could reach 97.83% within 30 min. This is because it decomposes under the catalysis of Pd^0^ to produce •OH, which oxidizes and degrades SMT.

After 180 min reaction, 42.4% of sulfamethazine would be degraded in the Fenton+H_2_+MIL-100(Fe) system, while in the MHACF-MIL-100(Fe) system the 97.8% of sulfamethazine could be removed in only 5 min. It would be thoroughly degraded in the next 0.5 h. The introduction of trace amount of Pd^0^ had a significant effect on the improvement of the degradation of the refractory organic.

As seen in Figure 7c, about 36% TOC was removed in the MHACF system. Organic intermediates such as 2-amino-6-methylpyrimidine-4-carboxylic acid, formic acid, CH_3_-CH=CH-CH_2_-CH_3_, p-aminobenzenesulfonic acid, CH_3_COOH, p-aminophenol, HOOC-COOH and 2-amino-4,6-dimethylpyrimidine could be detected in the aqueous solution. And the anion inorganic ions such as NH_4_^+^, NO_2_^−^, NO_3_^−^ and SO_4_^2−^ could also be detected in this system, as shown in Figure 7d. The results were similar with the previous research works [13,14,34]. The degradation of sulfamethazine could be mainly attributed to the oxidation of •OH.

### 3.5. The durability of MIL-100(Fe)

**Figure 8:**
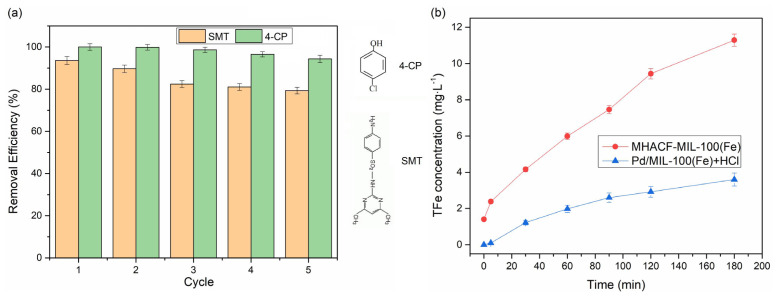
(a) The durability of catalyst in the MHACF-MIL-100(Fe) system, and the molecular structure of SMT and 4-CP; (b) total iron leakage without SMT and 4-CP in the MHACF-MIL-100(Fe) system and the control experiment. Experimental conditions: Fe^2+^ 24.6 μmol L^−1^, H_2_O_2_ 20 mmol L^−1^, pH 3.0, Pd/MIL-100(Fe) 2 g L^−1^, H_2_ 70 mL min^−1^, SMT 20 mg L^−1^ and 4-CP 12 mg L^−1^.

The 4-CP and SMT, which were the common refractory organics with different molecular structures, were selected as target pollutants, respectively. The former organic is an important chemical intermediate [29]. Both of them could be degraded by •OH [29]. The durability of the catalyst was investigated by repeatedly using the catalyst to degrade these two organic compounds. As displayed in Figure 8a, during the 5 reaction cycles, more than 94% of 4-CP could be stably removed. And at least 79% of SMT could be removed. According to the molecular structures displayed in Figure 8a, the molecular weight of SMT is higher than that of 4-CP. Therefore, 4-CP is more easily degraded by hydroxyl radical compared with SMT. However, with the increase of the reaction cycles, the degradation efficiency of these target pollutants gradually showed a downward trend.

By soaking MIL-100(Fe) in HCl (pH 3), as seen in Figure 8b, it was found that 3.6 mg L^−1^ TFe was dissolved after 3 h. And after 3 h operation of MHACF-MIL-100(Fe) system, there would be 11.29 mg L^−1^ of TFe was dissolved. The dissolved amount of TFe significantly exceeded its initial dosage. The Pd^2+^ was detected in the aqueous solution in both conditions. It could be speculated that the structure of MIL-100(Fe) may be changed during the operation of the MHACF system.

**Figure 9:**
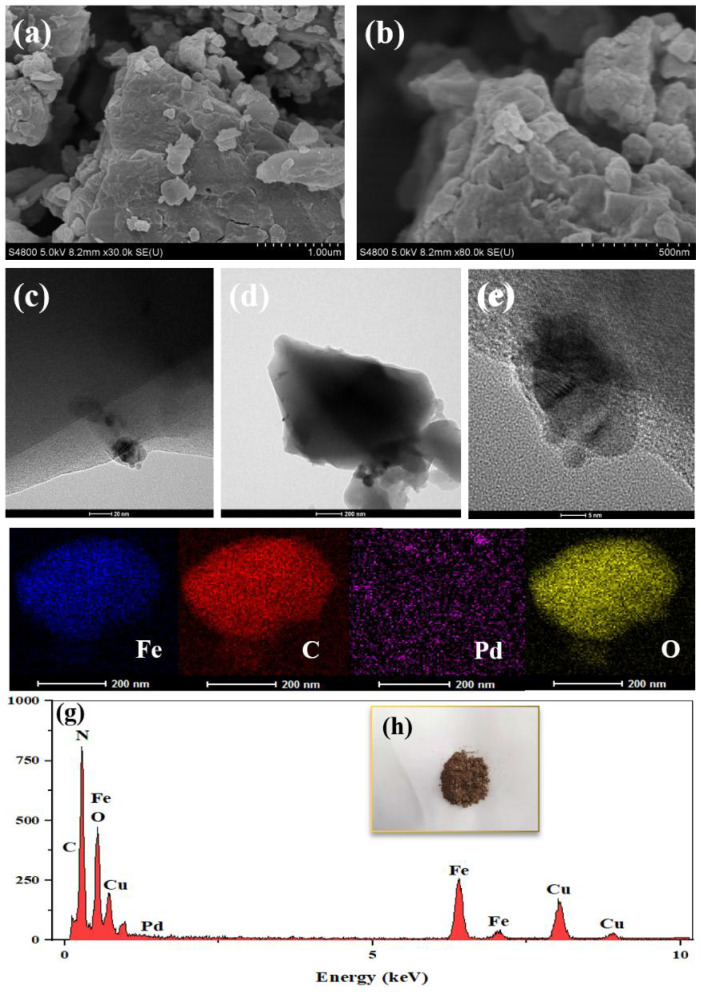
Pd/MIL-100(Fe) used on the degradation of SMT after five reaction cycles (a–e) SEM and TEM photographs at different magnifications; (f) elemental surface scans; (g) EDX spectra; (h) powder photographs.

**Figure 10:**
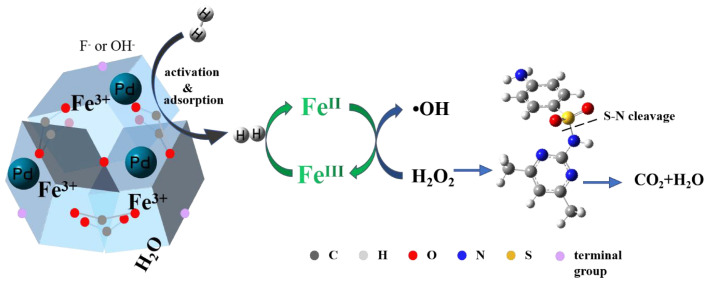
The mechanism of MHACF-MIL-100(Fe) system.

According to Figure 2, compared with that of Pd/MIL-100(Fe), the N_2_ adsorption-desorption curve of solid catalyst after 5 reaction cycles was still hysteresis-free loop class I isotherm though its specific surface area was slightly increased to about 1900 m^2^ g^−1^. And the pore size of the material still ranged from 0.5 nm to1.8 nm. The crystal structure of the material basically remained though the intensity of the peaks was slightly reduced. Combined with the electron microscope photographs exhibited in Figure 9, the basic morphology and elemental composition of MIL-100(Fe) was not significantly changed though Part of the Pd^0^ particle had fallen off. According to the results mentioned above, it could be confirmed that during the operation of the MHACF-MIL-100(Fe) system, the structure of the solid catalyst would be changed slightly, resulting in a small amount of total iron dissolution. The nano-Pd^0^ particle would gradually fall off during the reaction. Compared with the MIL-101(Cr) and NH_2_-MIL-101(Cr) used in our previous works [12], the MIL-100(Fe) had better durability. However, it was slightly less durable than that of UiO-66(Zr) and MOF-808(Zr) [13,14]. According to the soft and hard acid theory, this is because the high-priced metals have stronger acid resistance [12]. Firstly, we found the sole activity-MHACF-MIL-100(Fe) by differential experiments, and then we proved the presence of the main reactive oxygen ion -OH in the system by the probe capture method; then we explored the Fe^III^/Fe^II^ cycle in the MHACF-MIL-100(Fe) system, and found that the Fe^III^/Fe^II^ cycle in the MHACF-MIL-100(Fe) system is highly efficient, which makes this system have the ability of sustainable oxidation downgrade SMT. We then investigated the Fe^III^/Fe^II^ cycle in the MHACF-MIL-100(Fe) system and found that there is an efficient Fe^III^/Fe^II^ cycle in the MHACF-MIL-100(Fe) system, which makes this system have the ability to continuously oxidize and downgrade SMT, and in summary, through the accelerated reduction of hydrogen, it is possible to maintain the continuous production of •OH with the addition of only trace ferrous salts, and fundamentally reduce the production of iron sludge. Thus, continuous degradation of SMT in MHACF-MIL-100(Fe) system was achieved. The mechanism of MHACF-MIL-100(Fe) system was displayed in Figure 10.

## 4. Conclusion

MIL-100(Fe) was used to construct the MHACF system to degrade refractory organics at room temperature and pressure. By introducing hydrogen and Pd/MIL-100(Fe) catalyst into the classic Fenton system, based on the reduction of ferric enhanced by activated hydrogen, both SMT and 4-CP selected as the target pollutants could be rapidly degraded because hydroxyl radical could be incessantly generated. The production of iron sludge could be significantly reduced. The removal efficiency could be maintained at least more than 79% (sulfamethazine) and 94% (4-chloro phenol) after 5 reaction cycles though the Fe element was slightly dissolved, and the zero-valent palladium particle on the outer surface of the MIL-100(Fe) would also fall off. Therefore, it is essential to further explore stable MOFs materials and reasonable nano-Pd^0^ particle loading methods in future.

## Figures and Tables

**Table:** Determination conditions for liquid chromatography.

Organic	Mobile phase ratio	Flow Rate (mL min^−^^1^)	Sampling volume (μL)	Temperature (°C)	Wavelength (nm)	Reference
4-CP	Methanol/1% acetic acid solution = 65:35	0.5	20	30	278	^[15]^
SMT	Acetonitrile/water = 35:65	1	20	30	275	^[15]^
p-HBA	Methanol/0.1% acetic acid solution = 30:70	1	100	35	255	^[16]^
